# Nanoreactors for green catalysis

**DOI:** 10.3762/bjoc.14.61

**Published:** 2018-03-29

**Authors:** M Teresa De Martino, Loai K E A Abdelmohsen, Floris P J T Rutjes, Jan C M van Hest

**Affiliations:** 1 Eindhoven University of Technology, P.O. Box 513, 5600 MB Eindhoven, The Netherlands; 2 Radboud University, Institute for Molecules and Materials, Heyendaalseweg 135, 6525 AJ Nijmegen, The Netherlands

**Keywords:** catalysis, dendrimers, green chemistry, nanogels, nanoreactors, micelles, polymersomes

## Abstract

Sustainable and environmentally benign production are key drivers for developments in the chemical industrial sector, as protecting our planet has become a significant element that should be considered for every industrial breakthrough or technological advancement. As a result, the concept of green chemistry has been recently defined to guide chemists towards minimizing any harmful outcome of chemical processes in either industry or research. Towards greener reactions, scientists have developed various approaches in order to decrease environmental risks while attaining chemical sustainability and elegancy. Utilizing catalytic nanoreactors for greener reactions, for facilitating multistep synthetic pathways in one-pot procedures, is imperative with far-reaching implications in the field. This review is focused on the applications of some of the most used nanoreactors in catalysis, namely: (polymer) vesicles, micelles, dendrimers and nanogels. The ability and efficiency of catalytic nanoreactors to carry out organic reactions in water, to perform cascade reaction and their ability to be recycled will be discussed.

## Introduction

It is widely acknowledged that “the best solvent is no solvent”; however, running a reaction under neat conditions is very challenging from the points of view of mass transfer and temperature gradients [1–2]. Therefore, sustainable chemical technologies are often related to the use of a green non-harmful solvent [3], water. In principle, green chemistry refers to (1) the employment of raw material (substrates) in an efficient manner, (2) decreasing the resulting waste or undesired byproducts, and (3) using cheap and environment friendly solvents (i.e., water). Generally, using water as a solvent is an acceptable choice for green chemistry [4–6]. Indeed, water is attractive from both economic and environmental points of view, and is not taken into account when the E-factor (defined as mass ratio of waste to desired product) for a chemical process is determined [7–8]. This is to be true for chemical processes where the utility of water is limited to the work-up at the end of the process and not when used as a reaction medium. However, it should be noted that the utility of water as a reaction medium is the safest, but not the greenest choice. Unfortunately, most organic compounds and catalysts are not soluble in water, limiting its utility for most reactions [9–10]. For this reason, scientists across academia and industry have proposed many solutions in order to maximize the outcome of reactions (i.e., yields, enantioselectivities, etc.) in water and, thereby, harness its utility for further applications. The abovementioned issues are particularly relevant in the field of asymmetric catalysis, which besides overcoming catalyst compatibility also has to deal with cost issues [11–12]. Research on asymmetric catalysis has been mainly focused on performing catalytic reactions with high enantioselectivity and efficiency [13–14]. As a result, a wide range of chiral catalysts have been established [15–16]. Chiral catalysts are, however, not only incompatible with aqueous solutions, but also expensive due to the structural complexity of the ligands used and the usage of transition metals. Finding an approach to utilize chiral catalysts in water while minimizing their cost (i.e., recycling) is still a big challenge. In order to accomplish this, various strategies have been proposed and applied [17–19]. One significant, well-established and widely used strategy, is the use of site-isolated techniques, i.e., creating a separate micro environment [20–22] for catalysts to (1) allow their use in incompatible media, (2) to reduce their costs by recycling them, and (3) avoid any unfavorable environmental influences that might affect reaction yield and output [23–24]. Indeed, such a strategy proved to be advantageous for performing reactions in water and minimizing both reaction waste and cost [25–26].

Attempts to support homogeneous metal complexes onto organic or inorganic surfaces to facilitate their removal/extraction from the reaction mixture has proven to be successful [27–28]. In fact, the utility of catalytic supports has been fundamental to the concept of entrapping catalysts in organic nanodomains and bringing the notion of catalytic nanoreactors to light [29–30]. In recent years the use of nanocontainers/reactors wherein catalysts are entrapped and physically separated in an isolated compartment has appeared to be an excellent facile approach to enhance performance of reactions in water [31–34]. Pioneering examples in this field include small molecule host–guest containers such as cavitands [35–37], and calixarenes [38–39]. Besides these supramolecular cage structures compartmentalization can also be achieved in macromolecular nanoreactors. The advantage of employing these polymeric structures is their improved robustness and loading capacity, which makes recycling and efficient usage of catalytic species more achievable. Nanocompartments such as polymersomes [40], micelles [41], dendrimers [42], and nanogels [43–44] represent smart and compact devices to carry out reactions in aqueous media. Besides, their facile recyclability make them very suitable as nanoreactors for a multitude of applications in synthetic chemistry [24,31]. In a recent study the E-factors for different traditional coupling reactions used in the pharmaceutical industry were reported and compared to those achieved in micellar nanoreactors [45], showing for the latter a decrease of at least an order of magnitude, which underlines their considerable potential in green catalysis (Table 1).

**Table 1:** Representative comparison of E-factors (including the aqueous work-up), of a pharmaceutically relevant synthesis, carried out via a traditional and a micellar process [45].

reaction	E-factors in traditional process	E-factors in micelles

Heck coupling (300 g scale)	136	7.6
Suzuki–Miyaura (302 g scale)	83	8.3
Sonogashira coupling (57 kg scale)	37.9	7.0

In this review we will focus on the application of polymeric nanoreactors in green catalysis by highlighting their structure and ability to encapsulate and shield catalysts. Four different types of nanoreactors will be described, namely micelles, polymersomes, dendrimers and nanogels. The choice of discussing these nanoreactors stems from their accredited relevance in the field of catalysis and the significant number of examples published in literature. The advantageous aspects of these four classes of nanoreactors over non-supported homogeneous systems include: 1) the site isolation of reactive components (enabling cascade reactions), 2) the ability to convert hydrophobic substrates in water (under green conditions), and 3) the facile catalyst recovery. All these attractive features are covered in this review. Moreover, in this review we have not attempted to be comprehensive, but we rather want to illustrate the application potential of these nanoreactors with some illustrative examples of the most relevant classes of organic reactions (performed in water), which should interest both academia and industry.

## Review

### 1. Homogeneous vs heterogeneous catalysis

Catalysis, in general, is divided into two major types, homogenous and heterogeneous. In homogeneous catalysis catalyst and substrates are both present and molecularly dissolved in the same phase (typically a liquid phase) [46]. Homogeneous catalysis involves the use of biocatalysts (enzymes), organocatalysts and metal catalysts [47]. Catalysis is defined as heterogeneous when catalysts are in an aggregated state, and are thus in a different phase than the reactants [27,48]. Heterogeneous catalysts typically consist of a solid carrier, the so called “support”, on which catalytic sites are dispersed [49–50]. Homogeneous catalysis is generally performed under milder operative conditions than heterogeneous catalysis [51]. In fact, heterogeneous catalysts generally possess very high decomposition temperatures (above 100 °C) [52]. The presence of a solid phase often results in the formation of temperature gradients when using high temperatures, which leads to an increase in reactant diffusion and a consequent hampering of mass transfer [53]. Furthermore, the catalytic sites in heterogeneous catalysis are often not as well-defined as in homogeneous catalysis. Therefore, homogeneous catalysis usually results in better selectivity and less byproducts [54].

Although homogenous catalysis ensures high selectivity and a better reaction outcome, yet it is expensive (catalyst recycling is not always an option) and it requires the utility of harmful solvents, yielding high E-factors [53]. In order to lower the E-factor, water should be used in the work-up procedure and separation. It has to be pointed out, however, that the presence of water during the process and its purification afterwards, especially when coming from industrial wastes, poses stringent limitations from an economical and environmental point of view.

A good method for homogeneous catalysts separation and reuse is offered by the use of biphasic liquid–liquid systems. Recycling can be achieved in the reactor when the organic phase is sampled out, while the aqueous phase containing the catalyst is retained into the vessel, enabling for continuous processing. The main issue that has to be solved in such set-up is the tolerability of the catalyst to water (its solubility, its activity, etc.) [55]. A strategy to overcome this problem is the inclusion and confinement of the homogeneous catalysts into a host nano-architecture [56]. Compartmentalization enables catalyst segregation and shielding, and ensures its facile removal from the reaction mixture after the reaction has taken place [34]; this facilitates reactions to be performed in water followed by liquid–liquid separation of products and catalyst [22]. Moreover, shielding and segregation of catalysts in a nanoreactor facilitates one-pot tandem reactions that, in most cases, require two or more incompatible catalysts [22,57]. Catalyst confinement leads to a high local concentration of the substrate at the active site, which results in higher reaction rates and better conversion [9]. In this review we will highlight some typical nanoreactors that are used to accommodate homogeneous catalysts, holding promise in green organic synthesis. A division will be made between self-assembled nanoreactors, section 2, and covalent systems, section 3.

### 2. Self-assembled nanoreactors

Self-assembled nanoreactors are macromolecular architectures that are non-covalently assembled from their constituent building units [58–59]. Such nanoreactors allow for physical confinement of catalysts, shielding them from their surroundings [60]. Compartmentalization of catalysts in supramolecular nanoreactors is advantageous from kinetic (faster catalytic process) [61] and thermodynamic (lower transition state of reaction) [9] catalysis points of view. Segregation and isolation of catalysts inside nanoreactors guarantee, in most cases, a valuable platform for catalyst recycling [30]. In the following section we will discuss the utility of some of the well-established catalytic nanoreactors towards green(er) chemistry [62].

#### 2.1. Micelles

**Figure 1:**
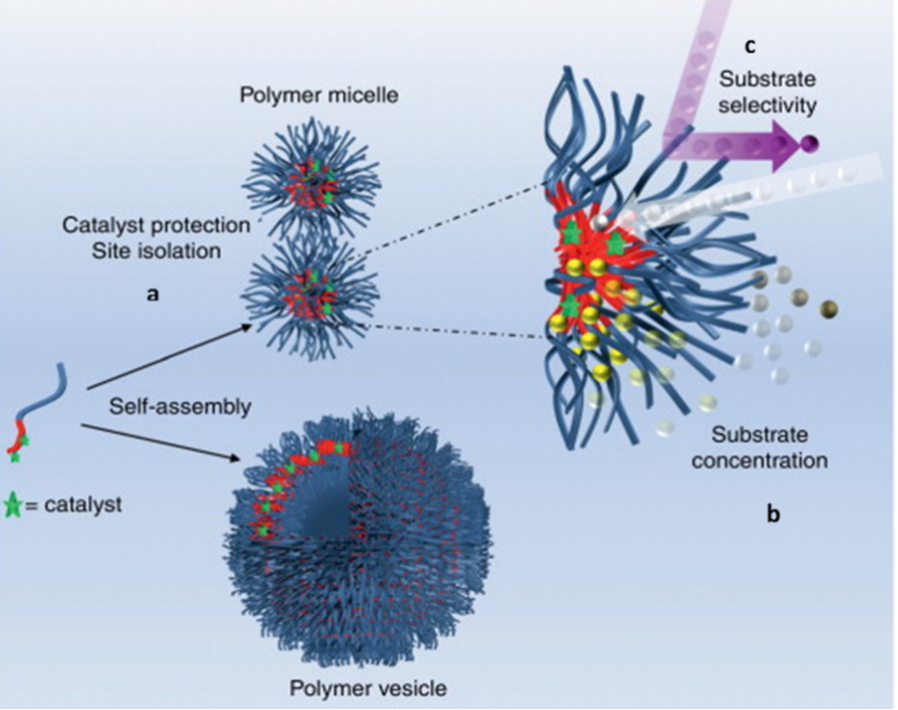
Assembly of catalyst-functionalized amphiphilic block copolymers into polymer micelles and vesicles. Characteristics of a nanoreactor system are shown using the polymer micelles including (a) the catalysts are protected and isolated from each other by the micellar shell, (b) substrates are effectively sequestered by the core from the surrounding environment, creating a highly concentrated environment for confined catalysis, (c) the nanostructure shell may regulate the access of substrates to the catalyst-containing micelle core. Reprinted with permission from reference [29].

Micelles are supramolecular architectures that are assembled of amphiphilic molecules [41]. Above the critical micellar concentration (CMC), surfactants with the appropriately designed hydrophilic head (neutral, anionic and cationic) and hydrophobic chain organize themselves in micelles [31]. Micelles have been extensively studied [9,32] and their utility as nanoreactors is well-established [41,58]. Various micellar morphologies can be obtained depending on the ‘packing parameter’ [56–61], which is defined as *p* = *v*/*a*_o_ *l*_c_, where *v* is the volume, *l*_c_ is the length of the hydrophobic chain and *a*_o_ is the optimal area of the head groups [62]. As a general rule, if *p* ≤ 1/3 spherical micelles are obtained, while cylindrical micelles, or the so-called worm-like micelles, form when 1/3 ≤ *p* ≤ 1/2. A typical micelle acquires a hydrophobic core that is able to accommodate hydrophobic catalysts, providing thermodynamic and kinetic control over chemical reactions [31]. Moreover, carrying out reactions in such a hydrophobic core leads to a concentration effect for hydrophobic substrates, which ensures higher reaction rates than those performed in bulk [63]. Besides, the structure of any micellar catalytic environment is governed by the arrangement of the amphiphilic molecules, creating, in many cases, a regioselective environment (Figure 1) that affects the outcome of some reactions [29].

Non-spherical, high aspect ratio micelles are preferred for catalysis as such structures provide large surface area where reactions could take place [64]. This has been particularly the case for dehydration reactions [24]. Due to the combination of the structures’ high aspect ratio and the hydrophobic effect, water could effectively diffuse away from the catalytic site, which enabled the enhanced formation of product. [40].

**Catalysis in micelles:** Micelles as nanoreactors have been extensively used in organic synthesis [31], allowing reactions in water [65] with better yields and easier catalyst recover [26] than traditional processes.

**Scheme 1:**
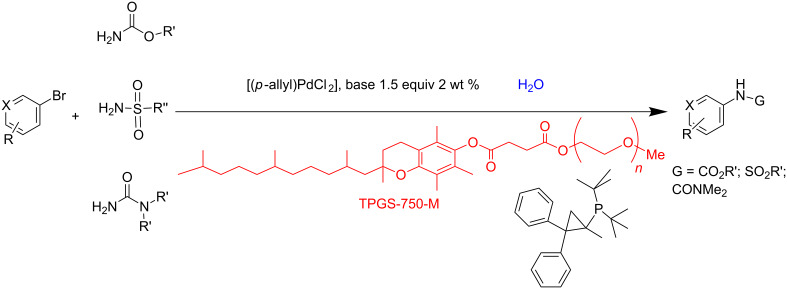
C–N bond formation under micellar catalyst conditions, no organic solvent involved. Adapted from reference [67].

Lipshutz and co-workers have successfully exploited micelles not only as nanoreactors, but as an outstanding platform for achieving greener organic reactions [26,65–66]. They have shown, for example, C–N cross-coupling reactions between heteroaryl bromides, chlorides or iodides and carbamate, sulfonamide or urea derivatives to be successfully realized in water using palladium-loaded TPGS-750-M (*dl*-α-tocopherol methoxypolyethylene glycol succinate) micelles (Scheme 1). Moreover, this micellar catalytic system allowed for catalyst recycling, minimizing the amount of the used organic solvent and generated waste [67].

The same group reported another interesting catalytic micelle system, which is based on PTS (polyoxyethanyl α-tocopheryl sebacate) [68]. Using PTS-based micelles, they showed the amination of allylic ethers in water (Table 2 and Table 3). The reaction of different ethers with naphthylmethylamine resulted in excellent yields (Table 2). Comparable yields were obtained when different amines reacted with *trans*-cinnamyl phenyl ether (Table 3). In both of the cases micelles were used to protect the very sensitive and unstable [Pd(allyl)Cl]_2_ intermediate from air.

**Table 2:** Reactions of allylic ethers **1a**–**e** with naphthylmethylamine^a^.

run	ether	time (h)	product	yield (%)

1	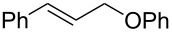 **1a**	20 (min)	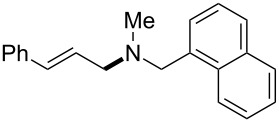	98
2	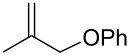 **1b**	1	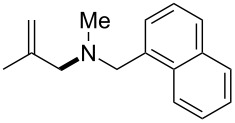	81
3	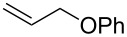 **1c**	5 (min)	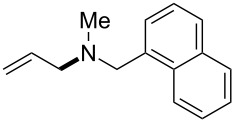	91
4	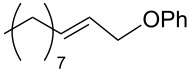 **1d**	1.5	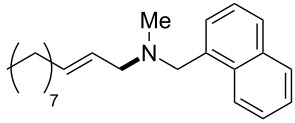 E:Z => 25:1	90
5	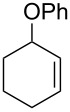 **1e**	5	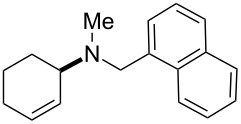	80

^a^Reactions were carried out under air at rt in 2 wt % PTS/water in the presence of [Pd(allyl)Cl]_2_ (0.5 mol %), bis[(2-diphenylphosphino)phenyl] ether (DPEphos, 1 mol %), ether (1 equiv), naphthylmethylamine (1.5 equiv), K_2_CO_3_ (1.5 equiv) and HCO_2_Me (4 equiv). Adapted from reference [68].

**Table 3:** Reaction of amines **2a**–**f** with *trans*-cinnamyl phenyl ether^a^.

run	amine	time (h)	product	yield^b^ (%)

A	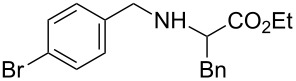 **2a**	7	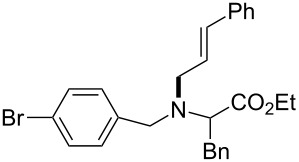	83
B	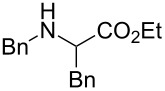 **2b**	2.5	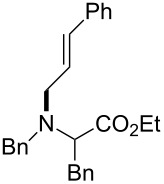	91
C	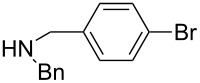 **2c**	14	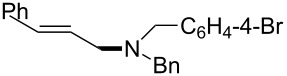	82
D	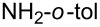 **2d**	2.5	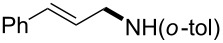	86
E	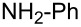 **2e**	2.5	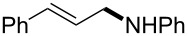	86 (9^c^)
F	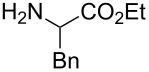 **2f**	2.5	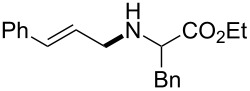	80 (6^c^)

^a^Reactions were carried out under air at rt in 2 wt % PTS/water in the presence of [Pd(allyl)Cl]_2_ (0.5 mol %), DPEphos (1 mol %), *trans*-cinnamyl phenyl ether (1 equiv), amine (1.5 equiv), K_2_CO_3_ (1.5 equiv) and HCO_2_Me (4 equiv). ^b^Isolated yields. ^c^Doubly allylated product. Adapted from reference [68].

Micelles were also used to perform cross-coupling between benzyl and aryl halides in water [65]. This reaction is known to result in very limited yields due to the undesired homo-coupling reaction between electron-rich and electron-poor benzyl bromides [69]. This draw-back has been circumvented by using Pd-catalytic micelles, which were assembled in water using TMEDA (tetramethylethylenediamine) as additive. TMEDA was used to stabilize the Pd catalyst by chelation and indeed, presence of TMEDA resulted in higher yield [65]. High catalytic efficiency of these Pd-catalytic micelles was also achieved while catalyzing reactions involving less reactive or sterically hindered species.

**Scheme 2:**
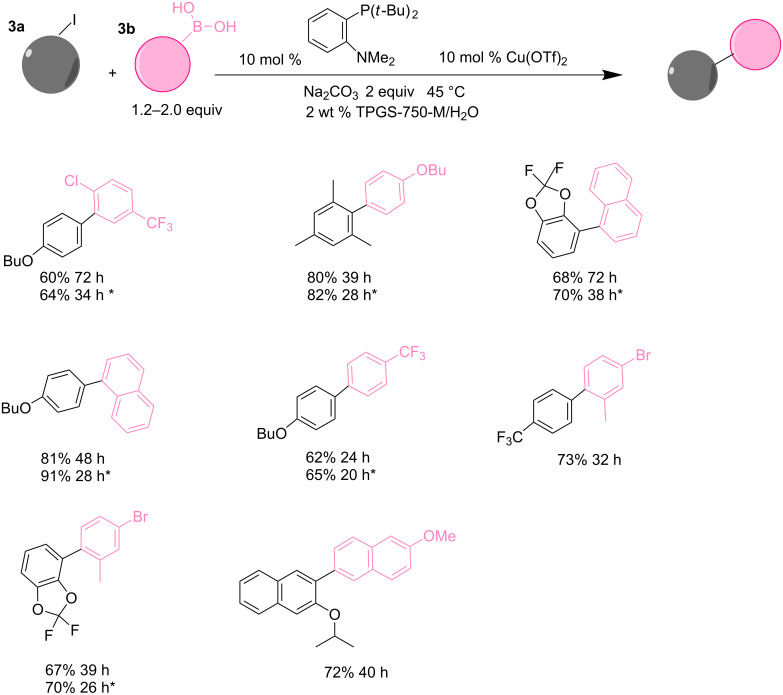
Suzuki−Miyaura couplings with, or without, ppm Pd. Conditions: ArI 0.5 mmol **3a**, Ar’B(OH)_2_ (0.75–1.00 mmol, 1.5–2.0 equiv) **3b**, *with 200 ppm of Pd(OAc)_2._ Adapted from reference [70]. Copyright 2016 American Chemical Society.

Handa et al. described a self-assembled TPGS-750M micelle (shown in Scheme 1), that allowed for copper-catalyzed Suzuki–Myaura coupling of aryl iodides (Scheme 2) [70]. When the reaction was conducted in inert atmosphere, no product was formed. However, the reaction was performed successfully in the presence of air, suggesting that the actual mechanistic pathway involved the formation of a P–(O)–N species on the ligand. The presence of traces of Pd was also needed in this process, as 200 ppm of Pd(OAc)_2_ worked like a co-catalyst being beneficial either for the reaction rate and the yields, and no product was observed without the Pd source. Furthermore, the recyclability of the catalyst was improved and the experiments could be repeated up to 5 runs with yields >90%. Contrary to the results obtained in bulk, using micelles resulted in higher yields even after catalyst recycling, providing a promising catalytic platform for these coupling reactions [70].

Lee et al. described an approach to perform catalysis in micelles based on rod–coil block copolymers [71]. Micelles were assembled from hydrophilic poly(ethylene oxide) (PEO) and hexa-*p*-phenylene, providing a platform for Suzuki reactions with the hydrophobic core acting as a suitable pocket for apolar aromatic guests [71–72]. With such a platform, full conversion was achieved at room temperature in water. Almost quantitative yields were observed when aryl chloride coupling was performed with arylboronic acids. This is indeed remarkable as aryl chlorides are generally not as reactive as aryl bromides or aryl iodides.

**Figure 2:**
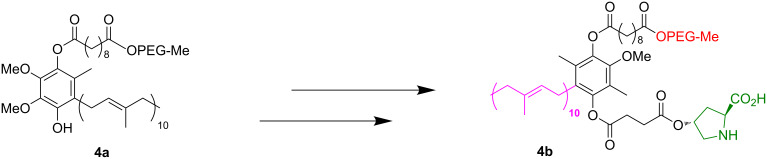
PQS (**4a**), PQS attached proline catalyst **4b**. Adapted from reference [26]. Copyright 2012 American Chemical Society.

Lipshutz and Ghorai developed a micellar system called PQS to perform aldol reactions in water [25]. As depicted in Figure 2, PQS (**4a**) has an OH moiety that allows for its linkage to the organocatalyst proline **4b**. Also, PQS has a lipophilic component that acts as a reaction solvent for hydrophobic dienes. The latter feature allows aldol reactions to take place efficiently in water.

The aldol reaction between cyclohexanone and *p*-nitrobenzaldehyde was chosen to verify the performance of this nanoreactor. PQS-proline and the analogous mixed diester derivative of 4-hydroxyproline were prepared and tested in this process. The aldol product was achievable only by using the proline compound **4b**, therefore different substrates were subsequently tested using 10 mol % of this catalyst in water at room temperature. The achievement of this study was not only on the stereoselectivity of the catalysts, but also on the substrate selectivity (Table 4): the preferred substrates are water-insoluble, suggesting that the reaction is occurring in the lipophilic pocket and not in water. The authors also demonstrated the ability of the PQS system to be recycled up to 10 times without loss in its catalytic activity.

**Table 4:** Representative PQS-proline **4b**-catalyzed reactions^a^:

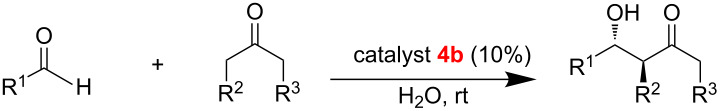					

entry	product	time (h)	yield (%)^b^	*anti*:*syn*^c^	ee^d^ (%)

1	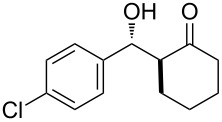	30	88	82:18	90
2	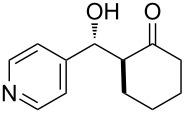	18	90	90:10	90
3	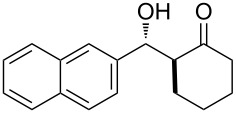	48	74	86:14	92
4	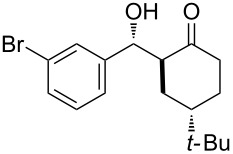	36	80	83:17	91
5	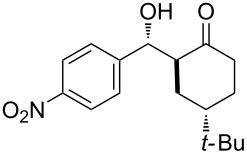	18	85	85:15	79
6	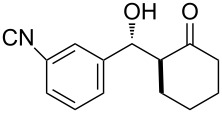	30	80	90:10	97
7	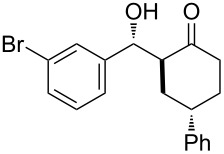	36	82	68:32	86
8	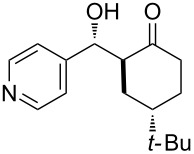	18	85	89:11	75
9	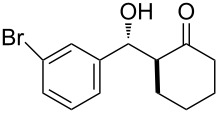	36	82	84:16	86
10	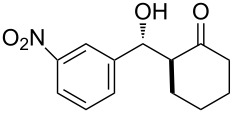	24	90	90:10	91

^a^The reactions were performed with aldehyde (0.01 mmol), ketone (0.5 mmol), and catalyst **4b** (0.01 mmol) at rt. ^b^Combined yield of isolated diastereomers. ^c^Determined by ^1^H NMR of the crude product. ^d^Determined by chiral-phase HPLC analysis for *anti*-products. Adapted from reference [26]. Copyright 2012 American Chemical Society.

Catalytic micelles were also prepared by O’Reilly et al. when a novel amphiphilic Sulfur–Carbon–Sulfur (SCS) pincer Pd catalyst together with a PAA (poly(acrylic acid)) based polymer self-assembled in water [32]. The catalytic activity of the nanostructures was compared to the results achieved with the small molecule analogues of the pincer Pd complex, in a Suzuki–Miyaura coupling. When the reaction of vinyl epoxide with phenylboronic acid was realized with 2% of pincer catalyst, the rate was 100 times higher for the water-based micellar system compared to the same reaction in organic solvent with the unsupported Pd-complex. A 100 times lower amount of catalyst was also loaded (0.02%), and still the reaction rate achieved was higher than the ones in organic media. This remarkable kinetic effect was attributed to two factors: 1) the small particle radius which increased the nanoreactor’s surface area, and 2) the creation of a more hydrophobic local pocket, as the catalyst was facing directly the hydrophobic membrane. Furthermore, the nanosystem also facilitated catalyst recycling by normal extraction.

#### 2.2. Polymeric vesicles

Polymeric vesicles or polymersomes are synthetic bilayered hollow architectures that are self-assembled from amphiphilic block copolymers [73]. The synthetic nature of polymersomes allows for facile tuning of their properties such as size [13,74], membrane permeability [75] and stability [76]. Various copolymers have been reported for polymersome formation such as poly(ethylene glycol)-*b*-polystyrene (PEG-*b*-PS) [14,77], polystyrene-*b*-polyisocyanopeptide (PS-*b*-PIAT)[21–22] and poly(*N*-isopropylacrylamide)-*b*-poly(ethylene oxide) (PNIPAM-*b*-PEO) [78]. The term “polymersomes” is derived from liposomes because of the structural resemblance. Compared to liposomes, polymersomes are mechanically robust vesicles and therefore considered to be highly attractive for nanoreactor applications [24,40]. Polymersomes comprise an aqueous lumen and hydrophobic membrane. Such hydrophilic and hydrophobic compartments are capable of accommodating hydrophilic (e.g., enzymes) or hydrophobic catalysts (e.g., metal catalysts) in their lumen or bilayer, respectively [28,79]. In an aqueous environment the hydrophobic membrane attracts hydrophobic substrates and brings them in proximity to the membrane-bound catalyst, leading to faster reaction rates. The presence of multicompartments in one system is interesting from a catalysis point of view as multistep cascades using incompatible catalysts can be achieved in one polymersome nanoreactor [22]. The compositional versatility of polymersomes thus allows for several applications in catalysis by encapsulating in or tethering catalysts to their compartments [33,80]. Polymersomes preserve and protect catalysts in their compartments improving, most of the times, catalytic activity and their performance in incompatible solvents such as water [21,24].

**Figure 3:**
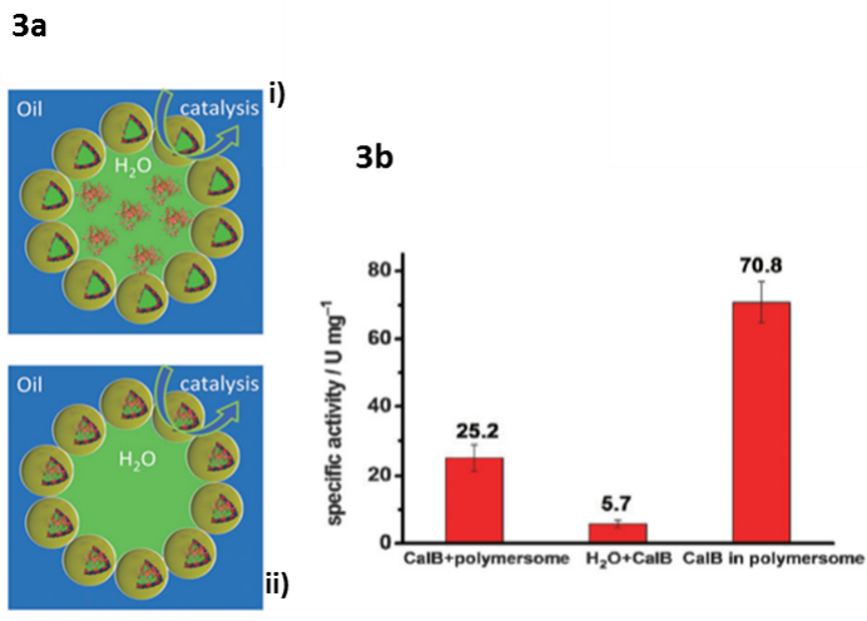
3a) Schematic representation of a Pickering emulsion with the enzyme in the water phase (i), or with the enzyme inside the polymersome lumen (ii). 3b) Chart of the specific activities of CalB dissolved in the water phase of the polymersome Pickering emulsion (left), CalB in a biphasic water/toluene system (middle,) and CalB encapsulated in the lumen of the polymersome Pickering emulsion (right). Adapted with permission from [79].

**Catalysis in polymersomes:** Polymersomes have been most often used as biocatalytic nanoreactors [22,81–83]. Polymersome nanoreactors were also employed in Pickering emulsions [83]. Pickering emulsions are emulsions stabilized by colloidal particles that adsorb at the water–oil interface. They are more stable than classical emulsions and do not require the usage of small molecule surfactants. This is a big advantage in downstream processing and product and catalyst recovery. The enzyme *Candida antarctica* lipase B (CalB) was encapsulated in the lumen of the polymersomes or in the Pickering emulsion water droplet. The esterification reaction of 1-hexanol and hexanoic acid was used to evaluate the catalytic performance of the CalB-loaded Pickering emulsions. Higher enzymatic activity was observed when CalB was encapsulated and the best results were achieved when the enzyme was in the lumen (Figure 3b), highlighting the advantage of enzyme compartmentalization and shielding. The explanation for this improved performance is the enlarged contact area between (hydrophobic) substrate and (water soluble) enzyme. Moreover, the system was recycled for at least 9 times without any loss in enzymatic activity.

**Scheme 3:**
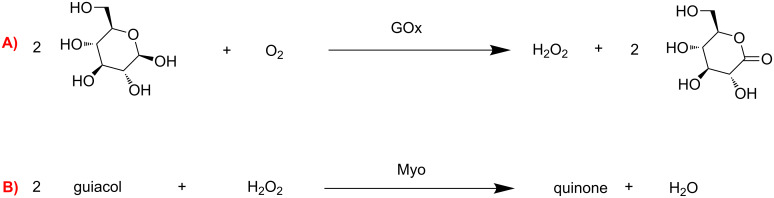
Cascade reaction with GOx and Myo. Adapted from reference [82].

Polymersomes have proven to be very useful for the performance of multistep catalytic conversions, in particular with enzymes [81]. Voit et al*.* studied the use of cross-linked pH sensitive polymersomes for the conversion of glucose in a tandem reaction [82]. The hydrophilic block of their polymersomes was PEG, and the hydrophobic block contained both poly[2-(diethylamino)ethyl methacrylate] (PDEAEM) which is pH responsive, and poly[4-(3,4-dimethylmaleimido)butyl methacrylate] (PDMIBM) as cross-linker. The activity of glucose oxidase (GOx) to convert glucose into D-glucono-δ-lactone and hydrogen peroxide was the first step of the reaction (Scheme 3A); subsequently, myoglobin (Myo) employed the hydrogen peroxide produced to oxidize guaiacol to quinone and water (Scheme 3B).

When the pH was below 7, the permeability of the cross-linked membrane allowed for substrate/product diffusion, but at basic pH the membrane collapsed and prevented any transport of small molecules. Two different activity tests were performed: 1) GOx and Myo were both entrapped inside the polymersome lumen; 2) GOx and Myo were individually incorporated into the polymersomes and mixed together in solution; in both of the cases the final product formation was monitored by UV–vis spectroscopy. The control over the pH allowed the regulation of the enzymatic cascade (no product was observed at pH 8 in both of the reactive systems), as the diffusion through the membrane was not possible. Moreover, the crosslinking enabled stabilization of the enzymes, which remained active also after 10 days.

**Figure 4:**
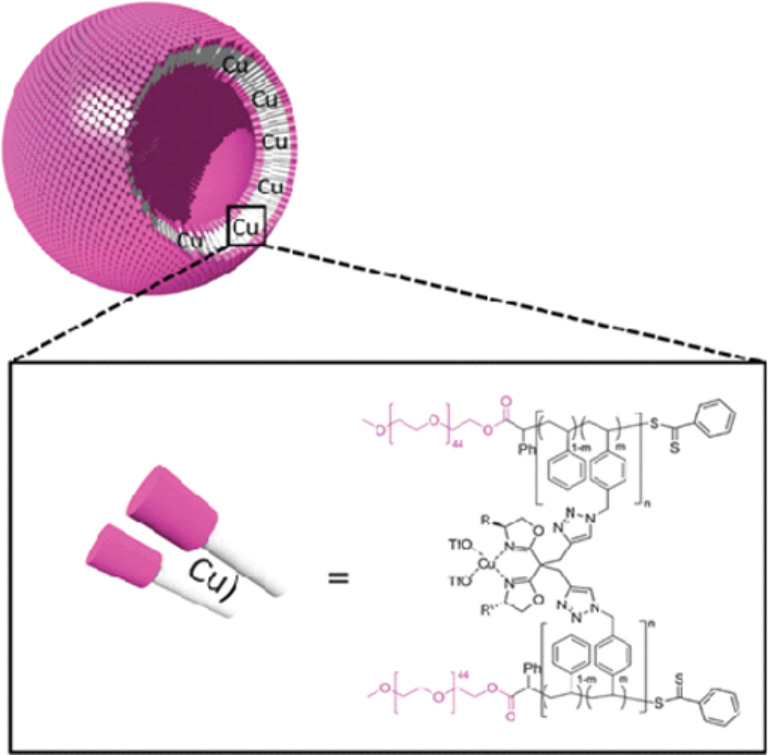
Cross-linked polymersomes with Cu(OTf)_2_ catalyst. Reprinted with permission from [15].

Polymersome nanoreactors have also been used to perform many types of non-enzymatic catalytic reactions, such as the proline-catalyzed asymmetric aldol reaction of cyclohexanone and 4-nitrobenzaldehyde [83]. Cross-linked polymersome nanoreactors were also used to perform asymmetric cyclopropanation reactions in water [15]. These products are highly desired intermediates in the preparation of agrochemicals and pharmaceuticals [84–86]. To perform cyclopropanation reactions in polymersomes, the membrane was cross-linked with bisoxazoline (BOX) ligands complexing the copper catalyst. Cyclopropanation reactions were efficiently performed in water, resulting in high yields and enantioselectivities, comparable to those when the reaction was carried out in organic solvent [80] (Figure 4).

As depicted in Table 5, substrate selectivity was observed when catalytic polymersomes were used, reasonably ascribed to a concentration effect, with more hydrophobic substrates leading to an increased local concentration around the catalyst in the hydrophobic membrane and as a consequence a higher reaction rate.

**Table 5:** Asymmetric cyclopropanation reaction of styrene derivatives and ethyl diazoacetate^a^.

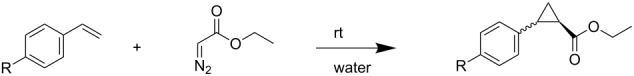							

entry	R	time (min)	load^b^ (%)	catalyst	conversion^c,d^ (%)	*trans*/*cis*^d^	ee *trans*^e^ (%)

1	H	120	10	C1	50^f^	73/27	60
2	H	10	10	C1	54	74/26	60
3	H	10	2	C1	12	72/28	60
4	H	10	10	C2	39	68/32	84
5	H	10	10	C3	43	59/41	34^g^
6	OMe	10	10	C2	93^h^	68/32	59^i^
7	Cl	10	10	C2	32^h^	75/25	53^i^
8	tBu	10	10	C2	67^h^	67/33	71

^a^Reactions carried out in 3.0 mL of Milli-Q water with 5.0 equiv of styrene and 1.0 equiv of ethyl diazoacetate. ^b^Catalyst loading in mol %. ^c^Conversion of ethyl diazoacetate into cyclopropane product. ^d^Determined by ^1^H NMR using triethylene glycol dimethyl ether as an internal standard. ^e^Determined by chiral GC. ^f^Polymersomes started to precipitate after 15 min. ^g^Configuration of the product was (1*S*,2*S*). ^h^Isolated yields. ^i^Determined by chiral HPLC. Adapted from reference [15].

Dergunov et al. reported on the design of a porous polymeric nanoreactor with a lipid bilayer for coupling reactions [87]. These nanocapsules were loaded with palladium catalysts and successfully used in Suzuki, Sonogashira and Heck cross-coupling reactions. Catalytic activity was compared to the activity of the freshly prepared free catalyst, and the palladium entrapment did not affect either the conversion or the yields of the reaction [28]. The catalyst immobilization also allowed facile Pd removal from the final product and catalyst recycling.

**Figure 5:**
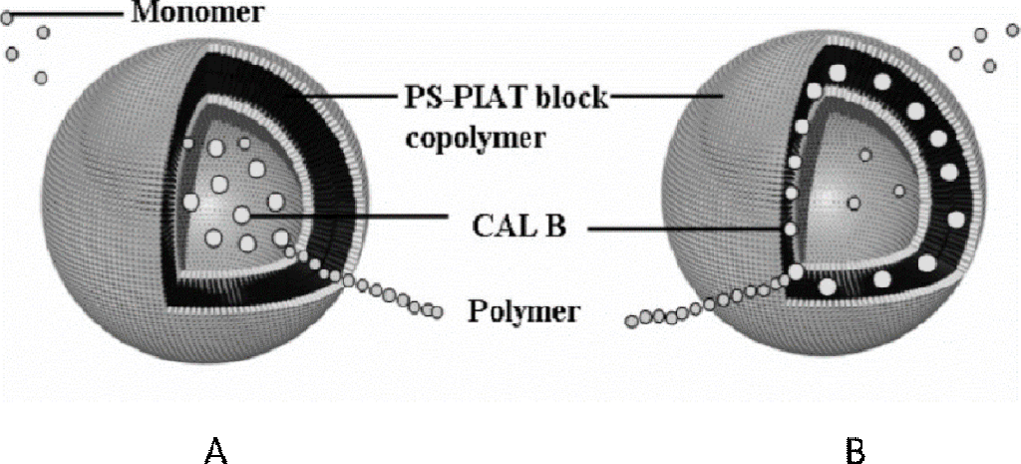
Schematic representation of enzymatic polymerization in polymersomes. (A) CALB in the aqueous compartment (B) CALB embedded in the bilayer. Reprinted with permission from [21]. Copyright 2007 American Chemical Society.

Polymeric nanoreactors were also used to perform ring-opening polymerization (ROP) in water. Nallani et al. reported on the enzymatic polymerization of lactones using CalB, which was immobilized in both the polymersome lumen and bi-layer [21]. Nanoreactors for ROP were prepared from polystyrene-polyisocyanopeptide (PS-PIAT) and CalB was incorporated within either the lumen or polymer membrane (Figure 5).

ROP is usually performed in organic solvent so that hydrolysis reactions can be avoided [17]. However, when nanoreactors were used, polymerization proceeded efficiently in water and without formation of any undesired products, providing a platform for aqueous ROP [21].

As shown in this section, polymersomes have been applied as a platform towards greener reactions [22,88], either by allowing reactions to be performed in water [21,83] or by providing a recyclable catalytic system [80]. As they contain both hydrophobic and aqueous compartments, they are especially useful for the immobilization of different catalysts, such as organocatalysts and enzymes that require different microenvironments for their optimal performance.

### 3. Covalent systems

#### 3.1. Dendrimers

Dendrimers are a class of highly branched molecules with high degree of symmetry [89]. They consist of different generations in which every generation is twice the molecular weight of the previous one. Dendritic architectures comprise three regions: a core, inner shell and outer shell [90]. The properties of dendrimers such as hydrophobicity can be tuned by varying their initial molecular components or the number of generations they possess [91–92]. They can assemble in a spherical shape, and within the three-dimensional structure, an interior void is present wherein to accommodate other molecules [93].

**Catalysis in dendrimers:** The controlled synthesis of dendrimers and their applications as nanoreactors and catalyst carriers have been extensively studied over the last decades [94–96]. Fan and co-workers incorporated a bis(oxazoline)-copper(II) complex in the hydrophobic core of a polyether dendrimer [11]. The copper catalytic complex was used to carry out asymmetric Mukaiyama aldol reactions. Although this system did not result in any substantial improvements in yields or enantioselectivities, it allowed for facile catalyst recovery and recycling.

Dendrimers were also used to encapsulate bimetallic catalysts to attain highly selective reactions [95,97]. The first successful attempt was reported by Chung and Rhee, in which they showed the encapsulation of a bimetallic Pt–Pd catalyst in a highly branched PMAM-OH dendrimer corona [93]. These catalytic dendrimers were employed in partial hydrogenation of 1,3-cyclooctadiene into cyclooctene. The utility of these dendrimers in hydrogenation reactions resulted in efficient reactions that proceeded with unprecedented selectivity of 99%. Moreover, this system is one of the first of bimetallic catalytic systems to be used for hydrogenation reactions in water.

**Figure 6:**
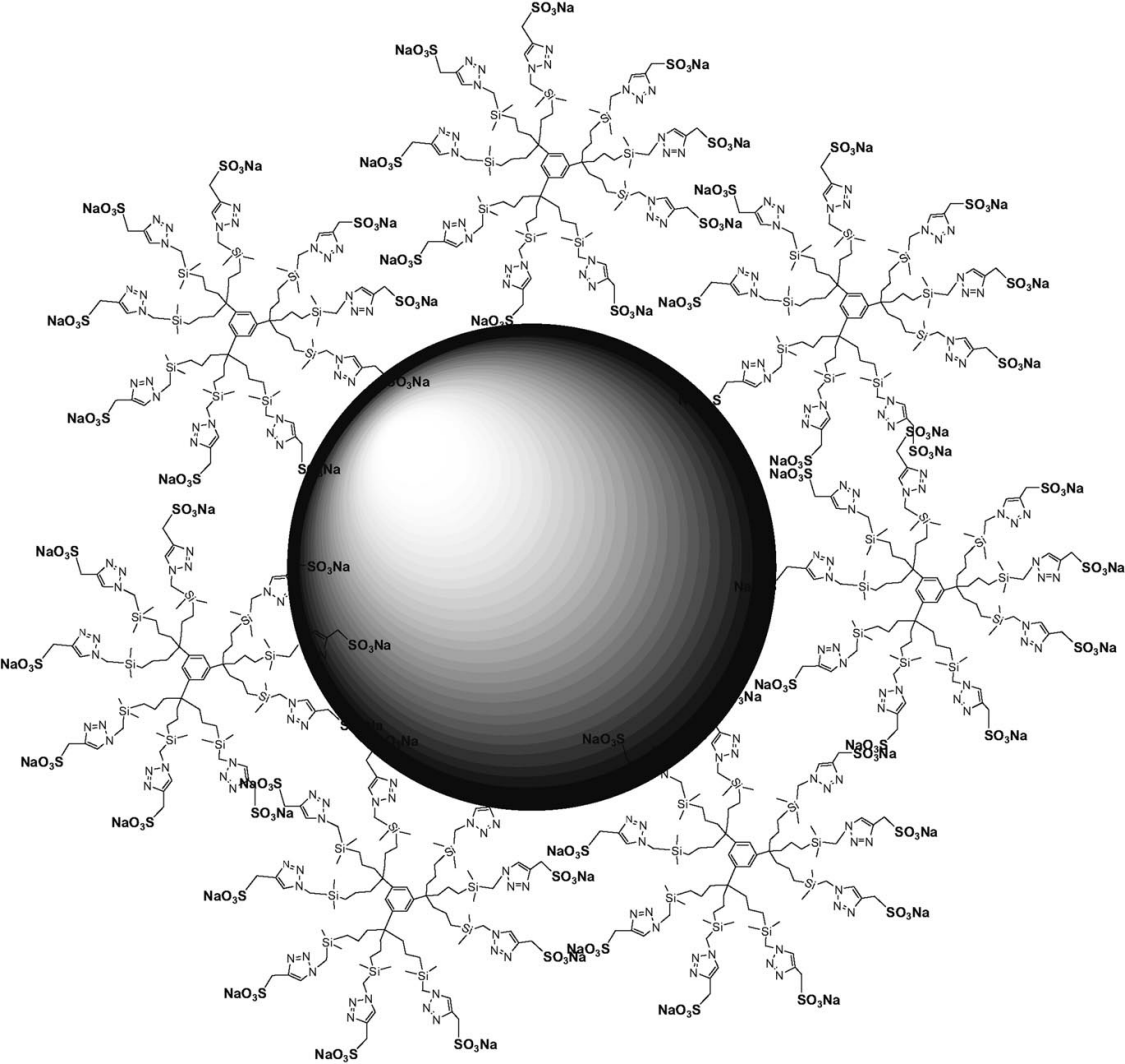
Representation of DSN-G_0_. Reprinted with permission from [100].

Water soluble dendrimer-stabilized nanoparticles (DSN) have been shown to be highly efficient in the catalysis of olefin hydrogenation and in Suzuki coupling reactions [98–99]. Ornelas et al. entrapped a palladium catalyst with dendrimers containing triazole groups (DSN) (Figure 6) [100].

The aim here was to provide a platform to perform hydrogenation in water. By using only 0.01% of palladium at room temperature, the hydrogenation of allyl alcohol was realized [101]. DSNs were recycled for up to 10 times without loss in activity. DSN nanoreactors were later shown to be utilized for catalysing Suzuki coupling reactions between PhB(OH)_2_ and PhX (X = I or Br) in water [100].

Other examples of water-soluble dendrimers are peptide- and glycol-based dendrimers [102–103]. As a result of their compositional versatility, they have been reported in many applications for biomedical engineering (e.g., glycopeptide dendrimers for drug delivery [104]).

The ability of peptide dendrimers to perform catalysis in an aqueous environment has also been investigated [105]. Many different libraries of peptide dendrimers have been used for biocatalytic applications, such as hydrolysis and aldolase reactions [105–108], showing their potential in green catalysis.

**Figure 7:**
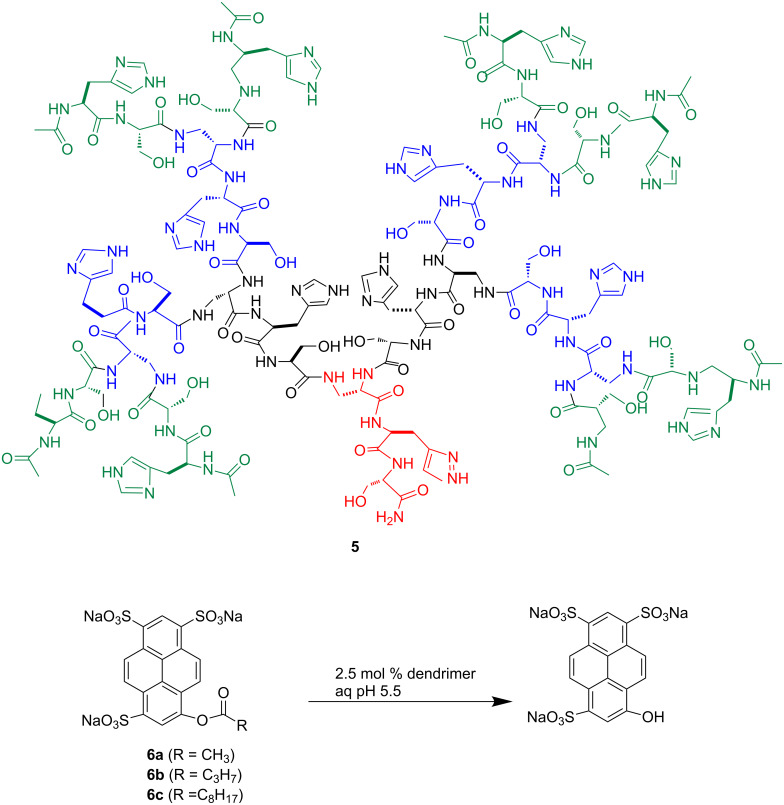
The multivalent esterase dendrimer **5** catalyzes the hydrolysis of 8-acyloxypyrene 1,3,6-trisulfonates **6a**–**c**. Reprinted with permission from [105].

Peptide dendrimers including aspartate, histidine and serine were utilized by Reymond et al. as catalytic esterase triad. Using fluorogenic 8-acyloxypyrene-1,3,6-trisulfonates as substrate (Figure 7) at the pH optimum of 5.5, triads’ activity was successfully demonstrated [107].

A noticeable rate enhancement was observed, related to a large apparent reactivity increase per catalytic site. Such an enhanced activity could be explained by the relatively high hydrophobic binding of the acyl group and the presence of histidine side chains that act as catalytic groups and as electrostatic substrate binding sites in their basic and acidic forms, respectively.

#### 3.2. Nanogels

Nanogels are hydrophilic polymer networks which can swell in the presence of water [109]. They can be crosslinked by either chemical bonds or physical methods, such as non-covalent interactions, entanglements and crystalline domains. The nanogels display excellent swelling behavior and are shape resistant [43,110]. Due to these unique properties they have mostly been studied as materials in biomedical applications such as controlled drug delivery [111]. Nanogels show promise as nanoreactors as they not only are colloidal stable particle in water but also can be prepared form a wide range of components and in many different sizes and shapes. They have been used for the templated synthesis of metal nanoparticles, via which the shape and size of the nanogel directed the formation of the corresponding particle with similar morphology [56,112]. The metal nanoparticle core is covered by polymeric brushes, the length and the grafting are important factors which can affect the reaction, as discussed in the following paragraph, and the easy manufacturing of metal nanoparticles makes the preparation of these core-brushes nanosystems suitable for many applications [113–115].

**Catalysis in nanogels:** Nanogels have intrinsic properties that make them well suited for green chemistry [116–117]. Water-compatible gels are usually based on poly(*N*-isopropylacrylamide) (PNIPAM), poly(*N*-vinylcaprolactam) (PVCL) or other water-soluble polymers [109]. For instance, PNIPAM is a thermo-responsive polymer, which has a lower critical solution temperature (LCST) of 32 °C. Above the LCST, individual polymer chains switch from a swollen coil configuration to a collapsed globular one, providing a nano-environment that is suitable for either hydrophobic or hydrophilic substrates [112]. Water forces PNIPAM brushes to become hydrophobic, acting as a suitable environment for most organic reactions [118]; it allows hydrophobic substrates to diffuse towards the encapsulated catalysts, leading to a concentration effect that directly contributes to an efficient aqueous reaction [119].

The preparation of catalytic nanocomposite hydrogels has been widely reviewed [56,114]. Several examples showing their utility as nanoreactors for various reactions such as coupling, oxidation and reduction reactions have been reported [43,114,118]. Wei et al. described a nanogel composed of poly(*N*-isopropylacrylamide) brushes grafted on Pd-NPs (Pd@PNIPAM) to carry out coupling reactions in water under mild conditions [120]. They showed highly efficient coupling of several hydrophobic aryl halides with phenylboronic acid, which in all cases resulted in yields above 70%. Moreover, the Pd@PNIPAM nanoreactors could be easily recycled thanks to the reversible phase-transition of the polymeric brushes [112].

**Figure 8:**
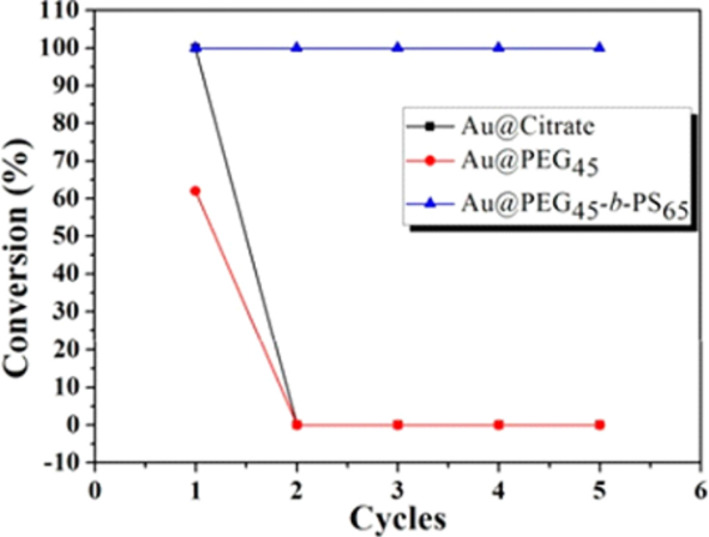
Conversion of 4-NP in five successive cycles of reduction, catalyzed by Au@citrate, Au@PEG and Au@PEG_45_-*b*-PS_65_. Reprinted with permission from [121]. Copyright 2015 American Chemical Society.

Que et al. reported the synthesis of gold nanoparticles (Au NPs) sheltered in PEG-PS nanogels for the reduction of 4-nitrophenol (4NP) [121]. Thiol functionalized PEG blocks were immobilized on Au NPs. PS segments improved the stability of the system and provided the necessary hydrophobic environment that is required to undertake the reduction reaction in water. The outcome of using Au@PEG-PS as nanoreactors was compared to those resulting from using both uncoated and PEG-coated Au NPs. While Au@PEG-PS resulted in quantitative conversions for 5 subsequent cycles, both uncoated and PEG_45_ coated Au NPs resulted in full and 61% conversions only for the first cycle, respectively. Recycling of uncoated and PEG_45_ coated Au NPs was not possible, highlighting the significance of the nanoreactor design (Figure 8).

Superior catalytic activity of Au@PEG-*b*-PS was observed in the reduction reaction of 4-nitrophenol to 4-aminophenol. The catalytic activity increased with the decrease in the chain length of the PS block. In addition, the high stability imparted by the PS layer endowed Au@PEG-*b*-PS with good reusability in catalysis without the loss of catalytic activity, and prevented from electrolyte-induced aggregation, making the system very attractive as nanoreactor.

Following on the previous work, He et al. synthesized cross-linked nanogels that were based on poly(acrylamide-*co*-acryl acid) (poly(AAm-*co*-AAc)) [117]. These nanogels were transformed into their catalytic counterparts by growing silver nanoparticles (Ag NPs) inside the cross-linked polymeric network. These catalytic nanogels were also used to catalyse the reduction of 4-nitrophenol to 4-aminophenol in water. The activity of these nanoreactors was tuned by varying the Ag NPs loading and the cross-linking density; higher activities were achieved by increasing the amount of Ag NPs loaded and decreasing the degree of polymer cross-linking. Such conditions facilitated the diffusion of water and substrates through the hydrogels and increased the probability of the reactant to be in contact with the catalyst (Ag NPs).

## Conclusion

In this review we have discussed the utility of supramolecular polymersomes, micelles, dendrimers and nanogels in catalysis. Over the past decades, many groups have demonstrated the specific features which make these nanoreactors an advantageous choice for chemical synthesis. In particular, they combine a high active surface area with a good dispersion in solution and therefore are ideal structures for facile diffusion of reactants. Furthermore, the compartments protect the catalyst from undesired interactions with the environment, which can be either the solvent, specifically water, or other catalytic species. As a result they allow reactions to proceed in water and often at room temperature, with excellent yields and selectivities, which traditionally can only be achieved by performing catalysed reactions in organic media. Moreover, although they are homogenously dispersed in the solvent, the nanoreactors are still large enough to be separated from the reaction mixture using standard filtration protocols. Therefore, they enable a facile purification and catalyst reuse.

This latter feature has potentially both an economic and environmental impact, deriving from a lower consumption of organic solvents, as lowering the E-factor in a process is a must for the modern chemical industry. Despite these many advantages, nanoreactors have not yet found widespread use in industry. A number of reasons can account for this. First of all, the construction of the nanoreactors is not always a cost-efficient process. Scalability and reproducibility in nanoreactor production also are key factors that have to be addressed. The recyclability and cost price should be improved to allow competition with existing heterogeneous and homogeneous processes. Furthermore, in most cases only model reactions have been studied. The improvement of a process that is highly relevant for industry would aid in a further acceptance of this technology by the end users. Another issue is that the specific characteristics of nanoreactors should be employed more effectively. Physical protection and separation of catalytic species will allow the performance of multistep conversions in one-pot reactors. This would then enable continuous flow processing, as intermediate work-up steps and solvent switching procedures can be prevented. Although this requires still much development, it is to be expected that in the near future nanoreactors will be key to a more sustainable production of fine chemicals.
